# Influence of dosimetry method on bone lesion absorbed dose estimates in PSMA therapy: application to mCRPC patients receiving Lu-177-PSMA-I&T

**DOI:** 10.1186/s40658-021-00369-4

**Published:** 2021-03-12

**Authors:** Julia Brosch-Lenz, Carlos Uribe, Astrid Gosewisch, Lena Kaiser, Andrei Todica, Harun Ilhan, Franz Josef Gildehaus, Peter Bartenstein, Arman Rahmim, Anna Celler, Sibylle Ziegler, Guido Böning

**Affiliations:** 1 Department of Nuclear Medicine, University Hospital, LMU Munich, Marchioninistrasse 15, 81377 Munich, Germany; 2 PET Functional Imaging, BC Cancer, 600 West 10th Avenue, Vancouver, BC V5Z 4E6 Canada; 3 Department of Radiology, University of British Columbia, 2775 Laurel Street, Vancouver, BC V5Z 1M9 Canada; 4 Department of Integrative Oncology, BC Cancer Research Centre, 675 West 10th Avenue, Vancouver, BC V5Z 1L3 Canada

**Keywords:** Radioligand therapy, mCRPC, PSMA, Lutetium-177, 3D dosimetry, Tumor dosimetry, OLINDA/EXM®, Voxel S value, Monte Carlo simulation

## Abstract

**Background:**

Patients with metastatic, castration-resistant prostate cancer (mCRPC) present with an increased tumor burden in the skeleton. For these patients, Lutetium-177 (Lu-177) radioligand therapy targeting the prostate-specific membrane antigen (PSMA) has gained increasing interest with promising outcome data. Patient-individualized dosimetry enables improvement of therapy success with the aim of minimizing absorbed dose to organs at risk while maximizing absorbed dose to tumors. Different dosimetric approaches with varying complexity and accuracy exist for this purpose. The Medical Internal Radiation Dose (MIRD) formalism applied to tumors assumes a homogeneous activity distribution in a sphere with unit density for derivation of tumor S values (TSV). Voxel S value (VSV) approaches can account for heterogeneous activities but are simulated for a specific tissue. Full patient-individual Monte Carlo (MC) absorbed dose simulation addresses both, heterogeneous activity and density distributions. Subsequent CT-based density weighting has the potential to overcome the assumption of homogeneous density in the MIRD formalism with TSV and VSV methods, which could be a major limitation for the application in bone metastases with heterogeneous density. The aim of this investigation is a comparison of these methods for bone lesion dosimetry in mCRPC patients receiving Lu-177-PSMA therapy.

**Results:**

In total, 289 bone lesions in 15 mCRPC patients were analyzed. Percentage difference (PD) of average absorbed dose per lesion compared to MC, averaged over all lesions, was + 14 ± 10% (min: − 21%; max: + 56%) for TSVs. With lesion-individual density weighting using Hounsfield Unit (HU)-to-density conversion on the patient’s CT image, PD was reduced to − 8 ± 1% (min: − 10%; max: − 3%). PD on a voxel level for three-dimensional (3D) voxel-wise dosimetry methods, averaged per lesion, revealed large PDs of + 18 ± 11% (min: − 27%; max: + 58%) for a soft tissue VSV approach compared to MC; after voxel-wise density correction, this was reduced to − 5 ± 1% (min: − 12%; max: − 2%).

**Conclusion:**

Patient-individual MC absorbed dose simulation is capable to account for heterogeneous densities in bone lesions. Since the computational effort prevents its routine clinical application, TSV or VSV dosimetry approaches are used. This study showed the necessity of lesion-individual density weighting for TSV or VSV in Lu-177-PSMA therapy bone lesion dosimetry.

## Background

The incidence of prostate cancer has been steadily increasing over the past decades in western populations [1, 2]. Patients with castration-resistant prostate cancer (mCRPC) typically present a large metastatic tumor burden in the bones [3]. Radioligand therapies (RLT) targeting the prostate-specific membrane antigen (PSMA) such as Lutetium-177-PSMA (Lu-177-PSMA) and Actinium-225-PSMA have shown promising results in patients ineligible for other therapies or have shown progress after receiving other systemic treatment options [4]. The clinical value of personalized dosimetry in RLT lies in a possible increase of the therapeutic window by limiting absorbed dose to organs at risk while maximizing absorbed dose to tumors. Thus, personalized dosimetry is indispensable for correlation with therapy response and patient outcome, enabling adjustments for subsequent therapy cycles. The first Lu-177-DKFZ-PSMA-617 absorbed dose estimates were published in 2015 [5]. Nonetheless, up to now, there are still few publications addressing the absorbed doses delivered to tumors after Lu-177-PSMA therapy [5–11]. While there is a clear definition of absorbed dose D as “the quotient of d$\overline{\varepsilon}$ by d*m*, where d$\overline{\varepsilon}$ is the mean energy imparted by ionizing radiation to matter of mass d*m*” in Report 85 of the International Commission on Radiation Units and Measurements (ICRU) [12], there are, however, different approaches for estimation of absorbed dose for internal radionuclide therapies, each with varying complexity and accuracy.

The use of pre-calculated organ-specific S values according to the Medical Internal Radiation Dose (MIRD) Committee formalism [13] has become more prevalent using the OLINDA/EXM® 2.0 software (HERMES Medical Solutions, Sweden) [14]. However, for the particular situation of tumor absorbed dose estimation, this approach relies on the unit density sphere model for calculation of tumor S values (TSV) that assumes homogeneous activity distribution within the spherical tumor and a tumor density of 1 g/cm^3^ (i.e., soft tissue). Thus, this fast and simple approach has limited applicability to bone lesions with higher densities and non-uniform activity distributions. Mass scaling of TSVs has been applied to include patient-specific density variations [15, 16], though the lesion-individual density in mCRPC patients may still limit the value of mass scaling of TSV. A three-dimensional (3D) voxel-wise dosimetry approach includes radionuclide-specific absorbed dose kernels or voxel S values (VSVs), which are pre-simulated for a specific tissue type and voxel size [17]. The use of VSVs accounts for heterogeneous activity distributions under the assumption of a homogeneous material and density [17]. Monte Carlo (MC) absorbed dose simulations based on SPECT/CT data include patient-individual, heterogeneous density, and activity distributions, yielding 3D voxel-wise absorbed dose estimations.

The aim of this work is to investigate various dosimetry techniques for accurate bone lesion absorbed dose estimation in Lu-177-PSMA therapy of mCRPC. The unit density sphere model for TSVs for volume of interest (VOI)-based dosimetry, and VSVs for different tissue types for 3D voxel-based dosimetry, without and with a tissue-specific density weighting were compared to patient-individual dosimetry by Monte Carlo simulations.

## Methods

### Patients

The study was conducted retrospectively on anonymized data and was approved by the local ethics committee of our institution. Fifteen patients with metastatic, castration-resistant prostate cancer (mCRPC) and pronounced metastases in the skeleton were included in this study. Table 1 presents the detailed patient characteristics. Patients received a first cycle of radioligand therapy using Lu-177-PSMA-I&T with activities of 7.4 GBq (10 patients) and 9.0 GBq (5 patients). The higher initial therapy activities were used in case of severe burden of bone metastases and/or presence of visceral metastases.

**Table 1:** Summary of patients being included. Previous treatment (1: yes; 0: no): OP surgery, RTx radiotherapy, AHT anti-hormonal therapy (including second line AHT with bicalutamide, enzalutamide, abiraterone acetate), CTx chemotherapy (docetaxel, cabazitaxel), Ra-223 radium-223 dichloride

Patient	Age	Activity (GBq)	PSA (ng/ml) prior to therapy	Gleason score	Previous treatment				
					OP	RTx	AHT	CTx	Ra-223
**1**	61	7.44	25.9	9	0	1	1	1	0
**2**	75	7.46	38.4	9	1	0	1	1	0
**3**	75	7.44	1070	8	1	1	1	1	1
**4**	78	9.04	570	9	0	0	1	1	0
**5**	62	7.47	848	-	0	1	1	0	0
**6**	59	7.47	5.38	7b	0	1	1	1	0
**7**	74	9.19	1696	-	1	1	1	0	0
**8**	63	7.46	149	8	0	1	1	1	0
**9**	82	7.44	20.2	9	1	1	1	0	0
**10**	70	7.42	127	9	1	1	1	1	1
**11**	75	9.05	436	9	0	1	1	1	0
**12**	49	9.00	121	9	1	1	1	1	1
**13**	64	7.47	1268	8	0	1	1	1	0
**14**	79	7.46	72.7	7b	0	0	1	0	0
**15**	73	9.04	19.6	9	1	0	1	1	0

### Image acquisition and reconstruction

Following the standard clinical routine imaging protocol of our institution, patients underwent quantitative Lu-177 SPECT/CT imaging (Symbia Intevo^TM^ T16 SPECT/CT, 3/8" crystal, medium-energy low-penetration collimator, Siemens Healthcare, Germany) at 24 h, 48 h, and 72 h post injection (p.i.). At least two SPECT bed positions were acquired in auto-contour mode followed by a low-dose CT. Image acquisition parameters included a 128 × 128 matrix with 64 angular steps and a duration of 5 s per step. These parameters were chosen as a compromise between covering the extended axial field of view (FOV) and patient comfort. The imaging energy window was centered at the energy of the upper photo peak of Lu-177 at 208 keV (width 15%). Quantitative SPECT reconstruction was performed with the clinically established Hermes Hybrid Recon v.2.1.1 reconstruction, which represents an ordered-subset ordinary-Poisson maximum a priori expectation maximization (OS-MAP-EM) reconstruction algorithm with a one-step late weighted quadratic penalty function and collimator-specific depth-dependent detector response modelling (16 MAP iterations, 8 subsets, Bayesian weight 0.01, HERMES Medical Solutions, Sweden) [18, 19]. CT-based attenuation correction and model-based scatter estimation as described by Sohlberg et al. [18] were used. The SPECT images were calibrated with a system-specific calibration factor, which was obtained using the same SPECT image acquisition and reconstruction parameters for a cylinder phantom (20 cm diameter), homogeneously filled with a known Lu-177 activity concentration [5, 20, 21].

### Image processing

All images were processed with PMOD (v4.005; PMOD Technologies LLC). Rigid co-registration of all CT and SPECT volumes was performed onto the SPECT/CT image data at 24 h p.i., which served as reference. An individual bone map and a whole-body VOI were derived from the reference CT by threshold-based segmentation (Hounsfield Unit (HU) threshold of 200 for bone map [3], HU threshold − 200 to − 100 for the whole body), and kidney VOIs were defined by manual delineation. To further segment individual bone lesions within the skeletal bone map, the semi-automatic k-means cluster segmentation of PMOD 3D tool was used on the 24-h SPECT [3]. All VOIs were copied to the co-registered SPECT data sets. Since image artifacts and noise impact voxel-wise fitting, time-activity curves were fitted in pre-defined VOIs to determine VOI-wise effective half-lives. VOI activities for the kidneys, tumor lesions, and remainder of the body (whole-body minus the kidneys and tumor lesions) were fitted using a mono-exponential fit model. A hybrid VOI/voxel-wise approach was used for generation of time-integrated activity images to partially maintain the voxel-wise activity distribution information. The time-integrated activity images per patient were generated with MATLAB (R2019b, The MathWorks, Inc. Natick, MA) based on the reference SPECT at 24 h p.i. and the individual VOI map:

1 $$ {\overset{\sim }{A}}^{voxel}=\frac{{\mathrm{A}}_{t=0}^{voxel}}{\lambda_{VOI}} $$

where $$ {\overset{\sim }{A}}^{voxel} $$ denotes the time-integrated activity per voxel, $$ {A}_{t=0}^{voxel} $$ is the activity at time point zero in a voxel, and $$ {\lambda}_{VOI}=\raisebox{1ex}{$\ln 2$}\!\left/ \!\raisebox{-1ex}{${t}_{1/2}$}\right. $$ uses the effective half-life obtained from mono-exponential fitting in the related VOI. $$ {A}_{t=0}^{voxel} $$ was computed as:

2 $$ {A}_{t=0}^{voxel}={A}_t^{voxel}\bullet {e}^{\lambda_{VOI}\bullet t} $$

with the time *t* being the exact time point of the individual 24 h p.i. SPECT acquisition.

### Dosimetry calculations

We investigated 7 different dosimetry approaches by utilizing the aforementioned time-integrated activity images and the reference CT of each patient.

#### MC method: Patient-specific Monte Carlo (MC) absorbed dose simulation

Patient-specific MC absorbed dose simulation accounts for the patient’s anatomy by using the geometry and density information from the patient’s CT image [22]. The radioactive decay, the interactions of the ionizing radiation with matter, and consequently the absorbed dose are simulated based on the patient-individual time-integrated activity distribution. Hence, MC absorbed dose simulations contain the highest level of complexity for modelling of radiation transport and interactions of ionizing radiation with matter with associated energy deposition among all other applied methods in this study. In concordance with inter alia Dieudonné et al. [23] and Grimes et al. [24], we considered MC dosimetry as the reference method assessing the other methods for bone lesion dosimetry. MC simulations in this study were performed using the GATE MC code version 8.2, based on GEANT4 version 10.5.1. This code has previously been validated for use in nuclear medicine therapies [25–27]. The radionuclide data were based on the Nuclear Data Sheets of Kondev et al. [28] and are the same as in OLINDA/EXM® [29]. A CT scan of a Gammex tissue characterization phantom (Gammex 467; Gammex Inc., Middleton, WI) using the same imaging parameters from the patient scans was performed, which confirmed the HU-to-density relationship of our CT device with that implemented in GATE. GATE converts HU-to-density values with internal tables based on Schneider et al. [22]. The time-integrated activity image of each patient was normalized with its total number of decays and used as the input for the simulations. The total number of 10^9^ primary decays per patient simulation was divided into 20 sub-simulations for parallel execution on separate CPUs to increase simulation speed (dual CPU system with 2 INTEL XEON 4114 CPUs, 10 cores each, 2.2 GHz, 192 GB RAM, running on Linux). The relative statistical uncertainty in the absorbed dose per voxel was calculated as described by Chetty et al. [30]. The voxel size of the simulation was (4.7952 mm)^3^ corresponding to the voxel sizes of the SPECT acquisitions. All particle range thresholds were set to 0.1 mm.

#### TSV method: Tumor S values (TSV) according to the unit density sphere model

The tumor S values from the uniform and unit density sphere model of OLINDA/EXM® 2.0 (HERMES Medical Solutions, Sweden) were used. This method represents the model with the lowest level of complexity and can be considered as the most simple and applicable method, yet clinically available. Since the total time-integrated activity per lesion and the lesion volume were known from the processing steps described above, the average lesion absorbed dose was calculated following the MIRD formalism [13] by multiplication of the tumor S value for the selected tumor volume with the tumor time-integrated activity. This approach is assuming that the lesion mass is comparable to the lesion volume at a tissue density of 1 g/cm^3^. TSVs are available for a limited number of sphere volumes/masses. Hence, the TSV per lesion was obtained by fitting the available TSVs within OLINDA/EXM®, and subsequent calculation of the TSV for the lesion mass *m* with the fit parameters (*TSV*(*m*) = 2.19 ∙ 10^−5^ ∙ *m*^−0.99^). This method includes solely the tumor self-dose [31] and is further based on the assumption that lesions were all of spherical shape with unit density and uniform activity distribution [32].

#### TSV_weighted_ method: TSV according to the unit density sphere model with additional lesion-individual density weighting

A simple method aiming to improve this absorbed dose estimate and to account for the tissue-specific tumor density is to convert the patient CT-image voxel-wise to densities using the HU-to-density conversion table, followed by the extraction of average lesion-individual density using the lesion VOI. The absorbed dose estimate is subsequently adjusted by weighting the lesion absorbed dose value *D*^*lesion*^ with the ratio of unit density and the average lesion-individual density ${\overline{\rho}}_{lesion}$, being equivalent to the mass scaling of S values [16]. This method takes into account the actual average lesion density ${\overline{\rho}}_{lesion}$ rather than assuming a fixed density for all lesions.

3 $$ {D}_{weighted}^{lesion}={D}^{lesion}\bullet \frac{1\ \raisebox{1ex}{$g$}\!\left/ \!\raisebox{-1ex}{${cm}^3$}\right.}{{\overline{\rho}}_{lesion}}. $$

#### VSV^soft^ method: Absorbed dose convolution model using voxel S values (VSVs) based on International Commission On Radiological Protection (ICRP) soft tissue

To account for the non-uniform activity distribution in 3D voxel-wise dosimetry, the use of VSVs for dosimetry has gained increasing interest [17]. For this purpose, GATE MC code was used for the simulation of Lu-177 VSVs using the voxel size of the time-integrated activity images, namely (4.7952 mm)^3^. The simulation used the soft tissue composition according to the ICRP [33, 34]. The central voxel of the ICRP soft tissue medium in a 51 × 51 × 51 matrix was set as Lu-177 source voxel, and 10^8^ primaries were simulated. The VSVs represent the absorbed dose distribution per decay such that when convolved with the time-integrated activity image this results in a patient-specific 3D voxel-wise absorbed dose map.

#### ${VSV}_{weighted}^{soft}$ method: Absorbed dose convolution model using VSVs based on ICRP soft tissue with additional density weighting

A limitation of the VSV^soft^ method was that the VSVs were simulated exclusively for soft tissue, and hence, the applicability for bone lesion dosimetry is hindered. Similar to the density weighting presented in the TSV_weighted_ method, it is possible to adjust for the different densities of the patient-individual anatomy and the density of the simulated VSVs. For this, the HUs of the patients’ CT were voxel-wise converted into density values. Consequently, the 3D voxel-wise absorbed dose map from the VSV^soft^ method is voxel-wise weighted with the ratio of the VSV density of ICRP soft tissue *ρ*_*ICRP*_ to the actual voxel density *ρ*_*voxel*_ [23]:

4 $$ {D}_{weighted}^{voxel}={D}^{voxel}\bullet \frac{\rho_{ICRP}}{\rho_{voxel}}. $$

#### VSV^soft+bone^ method: Absorbed dose convolution model using VSVs based on ICRP soft tissue and VSVs based on ICRP cortical bone

We extended the VSV^soft^ method by simulation of cortical bone VSVs using a standard ICRP cortical bone composition [33, 34] with the same simulation setup as for the ICRP soft tissue VSVs in the VSV^soft^ method. Similar to Lee et al. [35] who used multiple VSVs for regions with different tissues and densities, the combination of VSV^soft^ and VSV^bone^ was tested. For this, the patient’s bone map was used to distinguish between regions containing bone or soft tissue. The corresponding tissue-specific VSVs were applied in their respective regions. Subsequently, to obtain a total 3D voxel-wise absorbed dose map, the soft tissue 3D voxel-wise absorbed dose map (in soft tissue regions) and the cortical bone 3D voxel-wise absorbed dose map (in bone regions) are combined into a single image.

#### ${VSV}_{weighted}^{soft+ bone}$ method: Absorbed dose convolution model using VSVs based on ICRP soft tissue and VSVs based on ICRP cortical bone with additional density weighting

The skeleton itself is not merely composed of the cortical bone and shows a heterogeneous composition of tissues with varying densities. Therefore, to further account for the variations in bone composition, beyond the above-mentioned standard cortical model, a similar voxel-wise density weighting as in Eq. (4) is applied to the combined 3D voxel-wise absorbed dose map obtained from the VSV^soft+bone^ method in order to correct for differences in density per voxel.

### Comparisons

The TSV and TSV_weighted_ yield average lesion absorbed doses in agreement with the definition of average absorbed dose *D*^*av*^ in a chosen region of a specific tissue with total mass *m*_*t*_ as defined by Eq. 6.3 in the ICRU Report 86 [36]. To enable a comparison of this average absorbed dose per lesion *D*^*av*^ for the TSV approaches with the 3D MC voxel-wise absorbed dose maps, the average was formed accordingly, yielding ${D}_{MC}^{av}$. The percentage difference *PD*^*av*^ was calculated:

7 $$ {PD}^{av}=\frac{D_{method}^{av}-{D}_{MC}^{av}}{D_{MC}^{av}}\bullet 100 $$

To evaluate the 3D voxel-wise absorbed dose maps obtained from MC, VSV^soft^, ${\mathrm{VSV}}_{\mathrm{weighted}}^{\mathrm{soft}}$, VSV^soft+bone^, and ${\mathrm{VSV}}_{\mathrm{weighted}}^{\mathrm{soft}+\mathrm{bone}}$, the minimum absorbed dose within 25%, 50%, and 75% of the VOI volume per lesion was calculated, giving D25, D50, and D75.

For the assessment of the agreement of the different investigated 3D voxel-wise absorbed dose estimation methods, *PD*^*vox*^ was calculated on a voxel level for VSV^soft^, ${\mathrm{VSV}}_{\mathrm{weighted}}^{\mathrm{soft}}$, VSV^soft+bone^, and ${\mathrm{VSV}}_{\mathrm{weighted}}^{\mathrm{soft}+\mathrm{bone}}$ compared with MC:

9 $$ {PD}^{vox}=\frac{{\mathrm{D}}_{method}^{vox}-{D}_{MC}^{vox}}{D_{MC}^{vox}}\bullet 100 $$

Bland-Altman plots [37, 38] were used to compare the absorbed dose estimation methods.

## Results

**Fig. 1:**
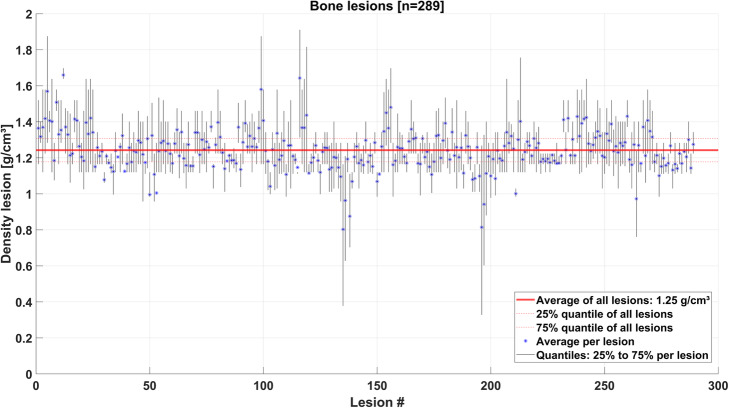
Density variation per lesion, given for all 289 bone lesions

In total, 289 bone lesions in the 15 mCRPC patients were evaluated. The segmented lesion volumes were on average 19.1 ml (range: 1.1 to 453.2 ml). The bone lesions were distributed within the whole skeleton. The majority of lesions were situated in the vertebrae (106), followed by the ribs (68), the extremities (64), and the pelvis (51). The average lesion density was 1.25 ± 0.11 g/cm^3^ (min: 0.80 g/cm^3^; max: 1.66 g/cm^3^), averaged over all 289 bone lesions. The density variation within each bone lesion is displayed for all lesions in Fig. 1.

### MC simulations

The overall simulation time per patient for the MC method was less than 4.5 h. The maximum relative statistical uncertainty in absorbed dose simulations was below 2.4 % for all voxels in all lesions and below 0.9 % on average over all lesion voxels. The maximum statistical uncertainty in the absorbed dose for the target region of ICRP soft tissue and ICRP cortical bone VSVs of the VSV^soft^, ${\mathrm{VSV}}_{\mathrm{weighted}}^{\mathrm{soft}}$, VSV^soft+bone^, and ${\mathrm{VSV}}_{\mathrm{weighted}}^{\mathrm{soft}+\mathrm{bone}}$ methods was below 3.2%. This was for the most distant voxel from the source voxel. The average over all target voxels was below 2.0%.

### Comparison of dosimetry methods

**Fig. 2:**
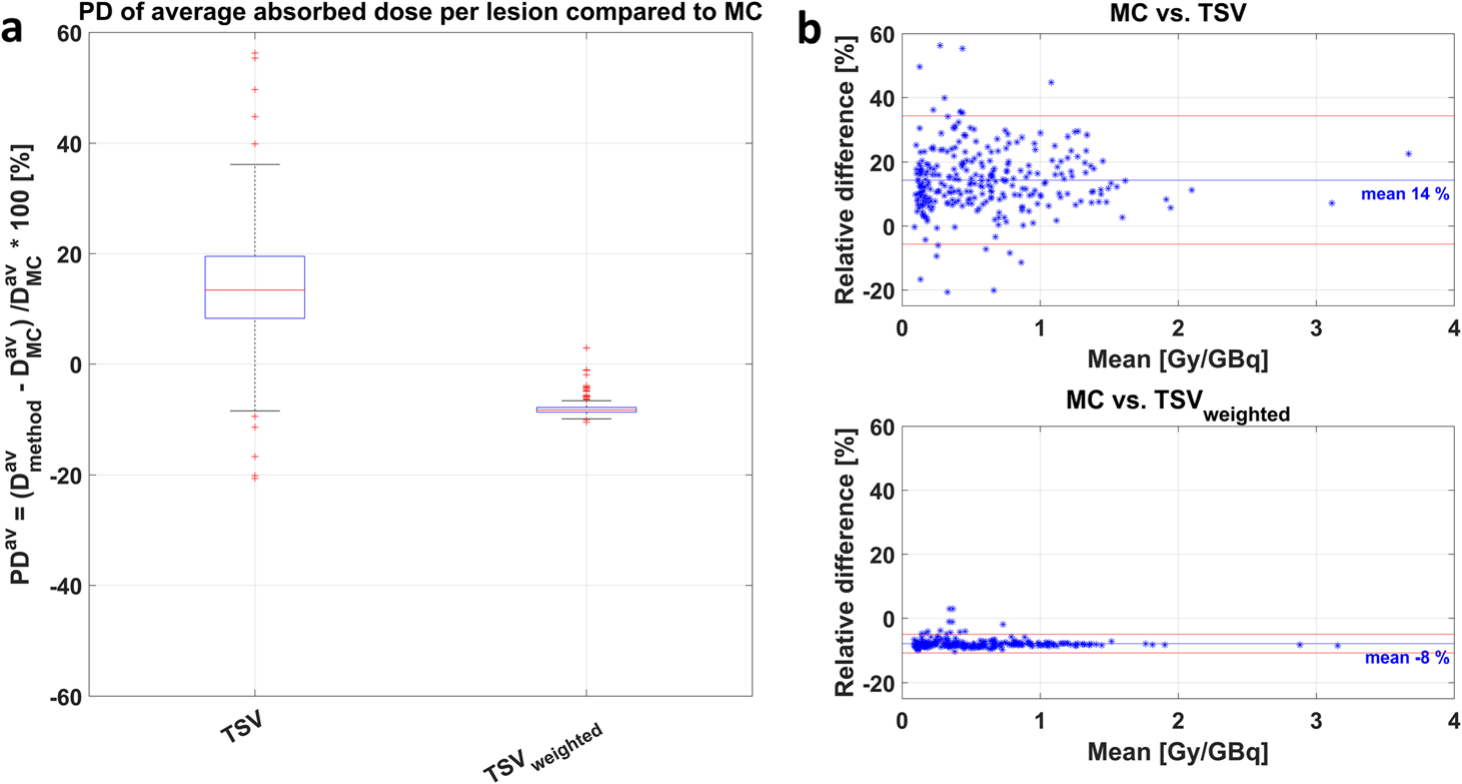
**a** Boxplot of *PD*^*av*^ per bone lesion of TSV and TSV_weighted_ compared to MC. **b** Bland-Altman plot

The percentage difference *PD*^*av*^ of average lesion absorbed dose estimates for the unaltered TSV was + 14 ± 10% (min: − 21%; max: + 56%) compared to MC, averaged over all lesions. The lesion-individual density weighting reduced the *PD*^*av*^ of TSV_weighted_ to − 8 ± 1% (min: − 10 %; max: − 3%). Figure 2a illustrates the decrease in range of *PD*^*av*^ for TSV_weighted_ compared to TSV, further supported by the Bland-Altman plot in Fig. 2b, showing the mean value of both methods compared to their relative difference.

**Fig. 3:**
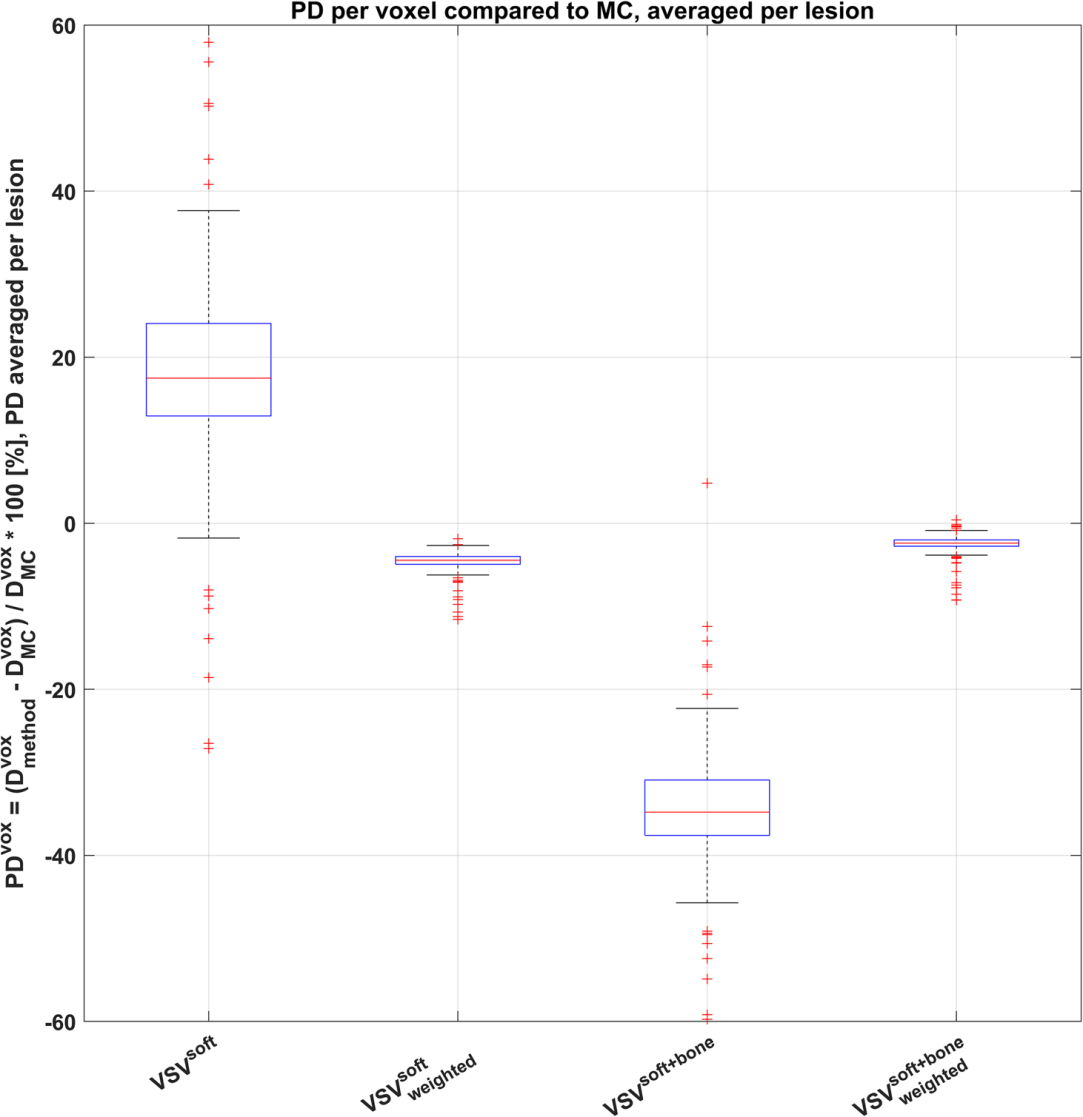
Boxplot of *PD*^*vox*^ of the 3D voxel-wise dosimetry methods compared against MC. *PD*^*vox*^ was averaged per lesion

The percentage difference (PD) of D25, D50, and D75 of VSV^soft^, ${\mathrm{VSV}}_{\mathrm{weighted}}^{\mathrm{soft}}$, VSV^soft+bone^, and ${\mathrm{VSV}}_{\mathrm{weighted}}^{\mathrm{soft}+\mathrm{bone}}$ methods compared to MC are given in Table 2, averaged over all lesions. The density weighting of VSV reduced the PD compared to the unweighted methods. The smallest PD of − 2% for D25, D50, and D75 was found for ${\mathrm{VSV}}_{\mathrm{weighted}}^{\mathrm{soft}+\mathrm{bone}}$. The evaluation on a voxel level revealed *PD*^*vox*^ of + 18 ± 11% (min: − 27%; max: + 58%) for VSV^soft^, averaged per VOI and over all lesions. This was reduced to − 5 ± 1% (min: − 12 %; max: − 2%) after voxel-wise density weighting for ${\mathrm{VSV}}_{\mathrm{weighted}}^{\mathrm{soft}}$. VSV^soft+bone^ showed *PD*^*vox*^ of − 34 ± 6% (min: − 60%; max: + 5%). ${\mathrm{VSV}}_{\mathrm{weighted}}^{\mathrm{soft}+\mathrm{bone}}$ showed the smallest *PD*^*vox*^ of − 2 ± 1% (min: − 9%; max: 0%). These observations are summarized in Fig. 3. The additional density weighting of ${\mathrm{VSV}}_{\mathrm{weighted}}^{\mathrm{soft}}$, and ${\mathrm{VSV}}_{\mathrm{weighted}}^{\mathrm{soft}+\mathrm{bone}}$, led to an overall smaller range of percentage differences than the associated method without weighting.

**Table 2:** PD in minimum absorbed doses to 25%, 50%, and 75% (D25, D50, D75) of the lesion VOI volume compared against MC. The PD was formed per lesion and then averaged over all lesion giving the presented value

Method	VSV^**soft**^ Mean ± SD	${\mathbf{VSV}}_{\mathbf{weighted}}^{\mathbf{soft}}$ Mean ± SD	VSV^**soft+bone**^ Mean ± SD	${\mathbf{VSV}}_{\mathbf{weighted}}^{\mathbf{soft}+\mathbf{bone}}$ Mean ± SD
**PD of D25 [%]**	**15 ± 14**	**− 4 ± 2**	**− 36 ± 8**	**− 2 ± 2**
**PD of D50 [%]**	**17 ± 11**	**− 4 ± 2**	**− 35 ± 6**	**− 2 ± 2**
**PD of D75 [%]**	**18 ± 10**	**− 5 ± 1**	**− 34 ± 6**	**− 2 ± 1**

**Fig. 4:**
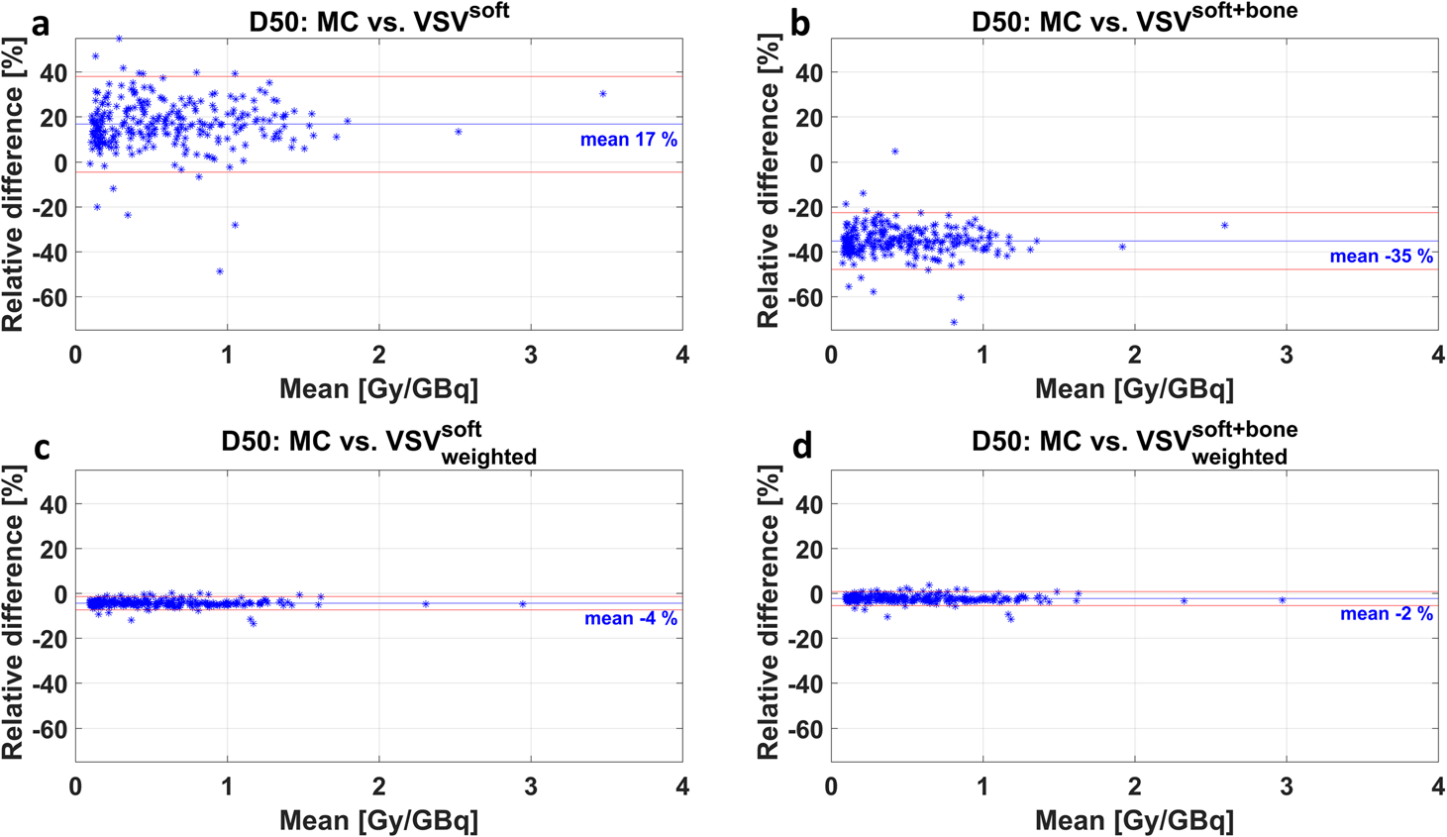
Bland-Altman plots of D50 compared against MC for **a** VSV^soft^, **b** VSV^soft+bone^, **c** ${\mathrm{VSV}}_{\mathrm{weighted}}^{\mathrm{soft}}$, and **d** ${\mathrm{VSV}}_{\mathrm{weighted}}^{\mathrm{soft}+\mathrm{bone}}$. The mean value of both methods was plotted against the relative difference of both methods. The blue line gives the mean relative differences and the red lines indicate the 95% limits of agreement

Figure 4 shows low bias for D50 compared to MC for the bone lesion absorbed dose estimates achieved with the density weighted ${\mathrm{VSV}}_{\mathrm{weighted}}^{\mathrm{soft}}$ (Fig. 4c) and ${\mathrm{VSV}}_{\mathrm{weighted}}^{\mathrm{soft}+\mathrm{bone}}$ (Fig. 4d). Furthermore, their corresponding limits of agreement and mean relative difference were the smallest with fewest outliers of all investigated 3D voxel-wise dosimetry methods. The Bland-Altman plots in Fig. 4a and b demonstrate the larger variations in lesion absorbed doses of the unweighted dosimetry methods compared to MC dosimetry.

**Fig. 5:**
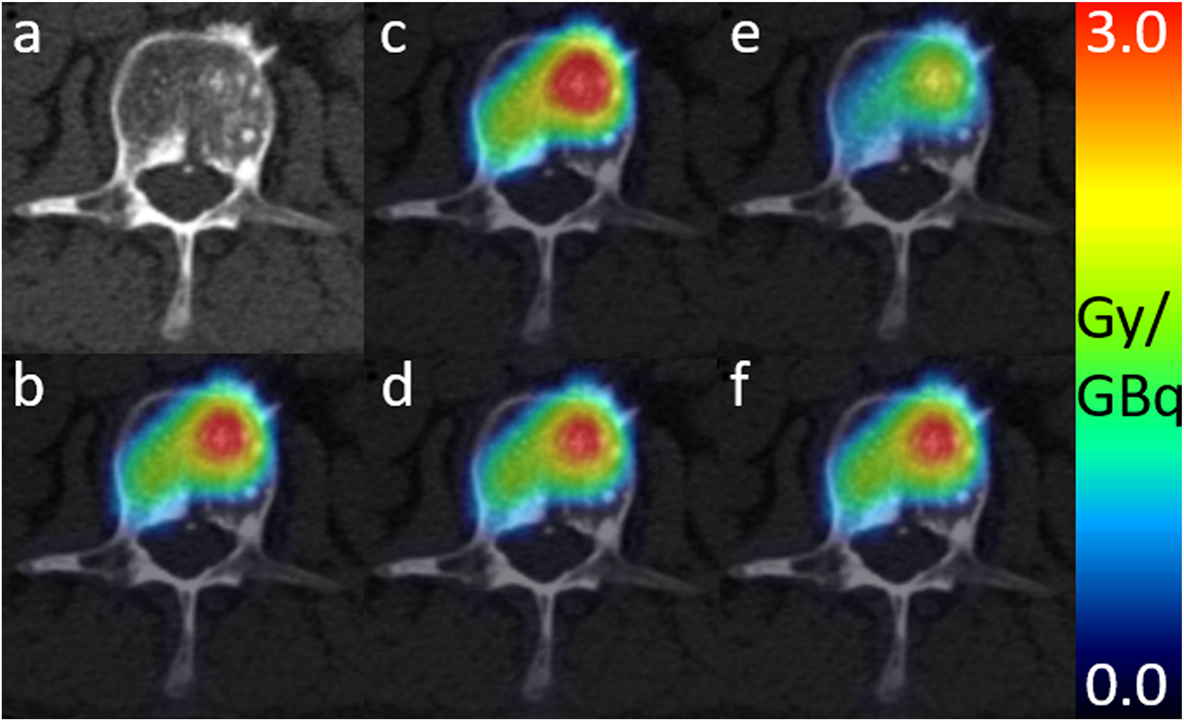
Patient example showing the transversal slice of 3D voxel-wise absorbed dose maps, fused with the patient’s CT image in **a**. Maps in units of Gy/GBq were achieved with methods: **b** MC, **c** VSV^soft^, **d** ${\mathrm{VSV}}_{\mathrm{weighted}}^{\mathrm{soft}}$, **e** VSV^soft+bone^, and **f** ${\mathrm{VSV}}_{\mathrm{weighted}}^{\mathrm{soft}+\mathrm{bone}}$. Average density of the displayed lesion was 1.20 g/cm^3^

Figure 5 visualizes a patient example showing the same transversal slice of 3D voxel absorbed dose maps from the 3D voxel-wise dosimetry methods fused with the corresponding slice of the patient`s CT (Fig. 5a). The 3D absorbed dose maps for the displayed bone lesion obtained from MC (Fig. 5b), ${\mathrm{VSV}}_{\mathrm{weighted}}^{\mathrm{soft}}$ (Fig. 5d), and ${\mathrm{VSV}}_{\mathrm{weighted}}^{\mathrm{soft}+\mathrm{bone}}$ (Fig. 5f) are comparable. The 3D absorbed dose map of VSV^soft^ (Fig. 5c) generally overestimates and VSV^soft+bone^ (Fig. 5e) underestimates the 3D absorbed dose map obtained from MC (Fig. 5b).

## Discussion

Patients with advanced mCRPC present with a considerably high tumor burden in the bone. Furthermore, osteosclerotic bone metastases can develop an increased number of osteoblasts leading to an elevated bone mass and increased density in the bone lesions [39]. Consequently, bone lesion absorbed dose estimates in Lu-177-PSMA therapy are affected by regional variations in bone tissue density, as observed in our investigations (Fig. 1). The absorbed dose estimates may depend on the strategy to account for these local changes. In this study, different techniques for VOI-wise and 3D voxel-wise dosimetry with varying complexity were compared. Simplified methods were tested against absorbed dose estimation by full Monte Carlo simulation. For this purpose, dosimetry results of 289 bone lesions of 15 mCRPC patients receiving their first cycle of Lu-177-PSMA-I&T therapy were assessed. To our knowledge, this study is the first to analyze and compare different dosimetric approaches for absorbed dose estimation in a high number of bone lesions in Lu-177-PSMA therapy.

The first method was based on the application of OLINDA/EXM®, which is widely clinically available and has been commonly used for dosimetry estimations in Lu-177-PSMA therapies [5–7, 9–11]. The percentage difference *PD*^*av*^ of average lesion absorbed doses compared to the MC average absorbed dose ${D}_{MC}^{av}$ ranged from an underestimation of − 21% to an overestimation by + 56%, yielding an averaged overestimation of + 14 ± 10% in all lesions. The broad spread of relative differences can partly be explained by the different assumptions made within this approach, i.e., spherical shape, uniform activity distribution, and unit density of the tumor. The latter may have the greatest impact for bone lesions with increased density. Using the VOI-based method TSV_weighted_, we hence attempted to correct for the different density of bone lesions compared to the unit density sphere model of TSVs by using the average lesion-individual density obtained from the patient’s CT scan. The mass scaling of the TSV with the lesion-individual average density addresses this assumption, yielding a reduced *PD*^*av*^ compared to MC as highlighted in Fig. 2. The spread of the *PD*^*av*^ of average lesion absorbed dose estimates was reduced to − 10 to − 3% with an average absorbed dose underestimation of − 8 ± 1%. This remaining difference may be associated to the assumptions that the tumor has only contributions of self-dose and is having a spherical shape in the TSV methods. Previous studies assessed the accuracy of absorbed dose estimation in soft tissue lesion against MC. Howard et al. [40] compared lesion absorbed dose estimates from the unit density sphere model of OLINDA/EXM® against MC simulation for Iodine-131 (I-131) radioimmunotherapy of lymphoma patients and concluded that the lesion shape has a minor impact when comparing the self-dose component. Their investigations revealed an absorbed dose underestimation compared to MC absorbed dose with a range of − 2 to − 31% PD, with average − 15 ± 8%. Grimes et al. [24] found good agreement of neuroendocrine tumor absorbed doses for Lu-177 from the unit density sphere model of OLINDA/EXM® and MC simulations with average percentage differences of − 3.8% ± 5.2%. Similar results with differences of − 5% were found by Divoli et al. [41], comparing absorbed doses of OLINDA/EXM® and MC for artificial spherical tumors in liver and lung. Our work assessed bone lesion absorbed dose estimation and the mass scaling of TSV_weighted_ with lesion-individual average density as described herein revealed *PD*^*av*^ compared to MC in the range of those reported in the literature [24, 40–42]. Pacilio et al. [43] investigated absorbed dose estimates for bone metastases of patients receiving Radium-223 (Ra-223) dichloride therapy. This publication used a fixed density of 1.4 g/cm^3^ for density weighting of the unit density sphere model of OLINDA/EXM®. If no lesion-individual density can be obtained using the patient CT image, this approach may result in more realistic values. However, the average lesion density for all 289 bone lesions investigated in this study was 1.25 ± 0.11 g/cm^3^, being lower than the proposed density of the skeleton of 1.4 g/cm^3^ [44]. The inter-lesion density variation displayed in Fig. 1 further supports the use of lesion-individual densities for mass scaling of TSV.

So far, 3D voxel-wise dosimetry calculations using VSVs were mainly applied in settings with heterogeneous activity distributions in homogeneous density distributions. For these implementations, a high agreement for tumor absorbed doses obtained from VSVs for soft tissue and MC simulation for soft tissue lesions was reported. Grimes et al. [24] reported only − 1.5 % ± 4.6% difference for Lu-177, and Dieudonné et al. [45] stated − 0.33% difference for Yttrium-90 (Y-90) and − 0.15% difference for I-131 for a hepatic tumor phantom. In general, VSV dosimetry calculations can account for heterogeneous activity distributions but not for density differences since they were simulated for a single homogeneous medium. For the majority of organs and lesions in the abdomen, only small density variations are assumed and a VSV^soft^ approach can therefore be safely used in the clinical setting. However, the assumption mentioned above has to be questioned in situations with large local tissue density changes. Thus, an adapted absorbed dose estimation approach becomes necessary for bone lesions in mCRPC patients. Based on our results for 3D voxel-wise absorbed dose calculations, we observed that both approaches, the utilization of single soft tissue VSVs (VSV^soft^) and of separate VSVs for soft tissue and bone (VSV^soft+bone^), reveal limitations in estimation of absorbed dose in bone lesions. Investigating the *PD*^*vox*^ revealed on average a strong overestimation by + 18 ± 11% (min: − 27 %; max: + 58%) for VSV^soft^. VSV^soft+bone^ on the other hand still showed limited capability of adequately estimating the absorbed dose per bone lesion; it exhibited a large underestimation of absorbed dose by − 34 ± 6% (min: − 60%; max: + 5%). These observations may be explained by the underestimated tissue density, which is an inherent characteristic of the soft tissue voxel absorbed dose kernel VSV^soft^, compared to the actual bone lesion density. Therefore, this underestimation of voxel density results in an underestimation of the voxel’s mass and consequently in an overestimation of the absorbed dose per voxel. On the other hand, VSV^soft+bone^ relies on the assumption that bone lesions consist merely out of the cortical bone, although a bone lesion can have different components and densities [46]. In this case, a larger mass than the actual lesion mass is assumed, and consequently, the observed absorbed dose is artificially smaller.

The VSV dosimetry methods with subsequent density weighting, as investigated in our study, seem to better address voxel-wise density changes and may therefore yield improved comparability with MC simulation. The proposed methods ${\mathrm{VSV}}_{\mathrm{weighted}}^{\mathrm{soft}}$ and ${\mathrm{VSV}}_{\mathrm{weighted}}^{\mathrm{soft}+\mathrm{bone}}$ led to significantly reduced *PD*^*vox*^ compared to Monte Carlo simulation, with an underestimation of on average − 5 ± 1% (min: − 12%; max: − 2%) and − 2 ± 1% (min: − 9%; max: 0%), respectively. These findings are supported by the Bland-Altman plots for D50 in Fig. 4c and d, obeying the smallest spread of data points and smallest mean relative difference compared to the MC method. Further, the majority of data points is within the 95% limits of agreement, given by the red lines. This observation is in concordance with Dieudonné et al. [23], who reported improved absorbed dose agreement for a density corrected VSV approach compared to full MC 3D voxel-wise dosimetry for three clinical cases with focus on soft tissue. Dieudonné et al. observed a lesion absorbed dose difference for a I-131-Tositumomab case of − 3.1%, an organ absorbed dose difference of maximum − 1.1% for a Lu-177-peptide case, and an organ absorbed dose difference of maximum + 0.8 % for a Y-90-microspheres case. Besides, Lee et al. [36] noted an overall improvement of whole-body absorbed dose estimates when introducing multiple tissue-specific VSVs, when compared to the utilization of a single tissue VSV. However, our results for bone lesion dosimetry indicate that the effect of additional density weighting onto a single VSV (${\mathrm{VSV}}_{\mathrm{weighted}}^{\mathrm{soft}}$ compared to VSV^soft^) outperformed the effect of adding multiple VSVs for various tissues without density weighting (VSV^soft+bone^ compared to VSV^soft^). In this work, VSVs were derived for a homogenous tissue. Hence, the application of absorbed dose kernel convolution approaches has limitations if neighboring voxels consist of different tissues. Due to the small maximum range of the β- particles of Lu-177 in soft tissue of 2 mm [15], and given the voxel size of (4.7952 mm)^3^ in this investigation, we expected this effect to be small when compared to the other effects investigated herein. In our study, we attempted to compensate for tissue differences with the proposed voxel-wise density weighting. Nevertheless, the magnitude of absorbed dose variations related to particle transport across tissue borders with respect to VSV methods requires further investigation.

3D voxel-wise dosimetry offers the visualization of regional variations in lesion absorbed dose estimates on a voxel level. The drawback of 3D voxel-wise dosimetry methods is that individual voxels can be influenced by image artifacts and noise. Further, the limited resolution of SPECT imaging leads to a spill-over of reconstructed activity between structures. Thus, the reconstructed 3D activity distribution does not fully represent a purely physiological activity distribution pattern and has to be interpreted with care. The development and potential amelioration to handle intra-skeletal partial-volume and spill-over compensation techniques should therefore be subject for future investigations. Within this work, we aimed at reducing the impact of the aforementioned effects by using quantitative SPECT reconstruction including distant-dependent point spread function of the detector and a hybrid VOI/voxel-wise approach to reduce the impact of noise and image artifacts on the determination of the time-integrated activity images which serve as an input for the 3D voxel-wise dosimetry methods. The applicability of density weighting is further limited to the CT resolution, and is thus not capable to account for heterogeneities on the sub-millimeter scale. In addition, co-registration of the 24 h, 48 h, and 72 h SPECT and CT images could potentially influence the absorbed dose estimates. This becomes relevant with regard to the outliers with small average lesion densities in Fig. 1, which represent lesions located in the ribs, with challenging co-registration due to breathing, patient’s motion, and less reproducible patient positioning between the image acquisitions from day to day. The different steps required for dosimetry include quantitative patient imaging, co-registration, segmentation, fitting, and time-integrated activity assessment, before any absorbed dose estimation can be made [47]. This work concentrated solely on this last step of absorbed dose estimation. The pre-processing was the same for all herein presented dosimetry methods, and thus, possible mistakes in the pre-processing would impact all methods equally.

## Conclusions

In our study of 289 bone lesions in mCRPC patients receiving Lu-177-PSMA-I&T therapy, the proposed voxel S value dosimetry approach with subsequent voxel-wise density weighting was associated with comparable absorbed dose estimates for bone lesions as obtained with full patient-individual Monte Carlo absorbed dose simulation. It therefore has the potential to enable routine patient-individual 3D voxel-wise dosimetry evaluations. Further, TSV approaches using lesion-individual average density for mass scaling provide fast and accurate average bone lesion absorbed dose estimates.

## Data Availability

Please contact author for data requests.

## Abbreviations

mCRPC: Metastatic, castration-resistant prostate carcinoma
PSMA: Prostate-specific membrane antigen
Lu-177: Lutetium-177
TSV: Tumor S value
VSV: Voxel S value
3D: Three-dimensional
MC: Monte Carlo
p.i.: Post injection
FOV: Field of view
VOI: Volume of interest
HU: Hounsfield Unit
MIRD: Medical Internal Radiation Dose
ICRP: International Commission On Radiological Protection
PD: Percentage difference
I-131: Iodine-131
Y-90: Yttrium-90
Ra-223: Radium-223
D25: Minimum absorbed dose to 25 % of lesion VOI volume
D50: Minimum absorbed dose to 50 % of lesion VOI volume
D75: Minimum absorbed dose to 75 % of lesion VOI volume
