# Correction to: Influence of dosimetry method on bone lesion absorbed dose estimates in PSMA therapy: application to mCRPC patients receiving Lu-177-PSMA-I&T

**DOI:** 10.1186/s40658-021-00382-7

**Published:** 2021-04-13

**Authors:** Julia Brosch-Lenz, Carlos Uribe, Astrid Gosewisch, Lena Kaiser, Andrei Todica, Harun Ilhan, Franz Josef Gildehaus, Peter Bartenstein, Arman Rahmim, Anna Celler, Sibylle Ziegler, Guido Böning

**Affiliations:** 1 Department of Nuclear Medicine, University Hospital, LMU Munich, Marchioninistrasse 15, 81377 Munich, Germany; 2 PET Functional Imaging, BC Cancer, 600 West 10th Avenue, Vancouver, BC V5Z 4E6 Canada; 3 Department of Radiology, University of British Columbia, 2775 Laurel Street, Vancouver, BC V5Z 1M9 Canada; 4 Department of Integrative Oncology, BC Cancer Research Centre, 675 West 10th Avenue, Vancouver, BC V5Z 1L3 Canada

**Correction to: EJNMMI Phys 8, 26 (2021)**

**https://doi.org/10.1186/s40658-021-00369-4**

Following publication of the original article [1], it was reported that due to a typesetting error some text was mistakenly introduced in the “MC method: Patient-specific Monte Carlo (MC) absorbed dose simulation” and “Comparison of dosimetry methods” sub-sections.

The erroneous text is highlighted in bold in the below passages and has been removed in the original article.

In the “MC method: Patient-specific Monte Carlo (MC) absorbed dose simulation” the affected sentence was:

A CT scan of a Gammex tissue characterization phantom (Gammex 467; Gammex Inc., Middleton, WI) using the same imaging parameters from the patient scans was **perfMC method: Patient-specificormed**, which confirmed the HU-to-density relationship of our CT device with that implemented in GATE. GATE converts HU-to-density values with internal tables based on Schneider et al. [22].

The corrected sentence reads:

A CT scan of a Gammex tissue characterization phantom (Gammex 467; Gammex Inc., Middleton, WI) using the same imaging parameters from the patient scans was performed, which confirmed the HU-to-density relationship of our CT device with that implemented in GATE. GATE converts HU-to-density values with internal tables based on Schneider et al. [22].

In the “Comparison of dosimetry methods” sub-section the affected sentence was:

The additional density **wePatient example showing the transversal slice ofighting** of ${VSV}_{weighted}^{soft}$ and ${VSV}_{weighted}^{soft+ bone}$, led to an overall smaller range of percentage differences than the associated method without weighting.

The corrected sentence reads:

The additional density weighting of ${VSV}_{weighted}^{soft}$ and ${VSV}_{weighted}^{soft+ bone}$, led to an overall smaller range of percentage differences than the associated method without weighting.

The original article has been updated.
